# Heterogeneity of Colorectal Cancer Risk Factors by Anatomical Subsite in 10 European Countries: A Multinational Cohort Study

**DOI:** 10.1016/j.cgh.2018.07.030

**Published:** 2019-06

**Authors:** Neil Murphy, Heather A. Ward, Mazda Jenab, Joseph A. Rothwell, Marie-Christine Boutron-Ruault, Franck Carbonnel, Marina Kvaskoff, Rudolf Kaaks, Tilman Kühn, Heiner Boeing, Krasimira Aleksandrova, Elisabete Weiderpass, Guri Skeie, Kristin Benjaminsen Borch, Anne Tjønneland, Cecilie Kyrø, Kim Overvad, Christina C. Dahm, Paula Jakszyn, Maria-Jose Sánchez, Leire Gil, José M. Huerta, Aurelio Barricarte, J. Ramón Quirós, Kay-Tee Khaw, Nick Wareham, Kathryn E. Bradbury, Antonia Trichopoulou, Carlo La Vecchia, Anna Karakatsani, Domenico Palli, Sara Grioni, Rosario Tumino, Francesca Fasanelli, Salvatore Panico, Bas Bueno-de-Mesquita, Petra H. Peeters, Björn Gylling, Robin Myte, Karin Jirström, Jonna Berntsson, Xiaonan Xue, Elio Riboli, Amanda J. Cross, Marc J. Gunter

**Affiliations:** ∗ Section of Nutrition and Metabolism, International Agency for Research on Cancer, Lyon, France; ‡ Department of Epidemiology and Biostatistics, School of Public Health, Imperial College London, London, United Kingdom; § Le Centre de recherche en Epidémiologie et Santé des Populations, Faculte de Médecine, University Paris-Sud, Faculte de Médecine, Université de Versailles Saint-Quentin-en-Yvelines, INSERM, Université Paris-Saclay, Villejuif, France; ‖ Gustave Roussy, Villejuif, France; ¶ Department of Gastroenterology, Bicêtre University Hospital, Assistance Publique des Hôpitaux de Paris, Le Kremlin Bicêtre, France; # Division of Cancer Epidemiology, German Cancer Research Center, Heidelberg, Germany; ∗∗ Department of Epidemiology, German Institute of Human Nutrition, Potsdam-Rehbrücke, Germany; ‡‡ Department of Epidemiology, Nutrition, Immunity and Metabolism Start-up Laboratory, Potsdam-Rehbrücke, Germany; §§ Department of Research, Cancer Registry of Norway, Institute of Population-Based Cancer Research, Oslo, Norway; ‖‖ Department of Medical Epidemiology and Biostatistics, Karolinska Institutet, Stockholm, Sweden; ¶¶ Genetic Epidemiology Group, Folkhälsan Research Center and Faculty of Medicine, University of Helsinki, Helsinki, Finland; ## Department of Community Medicine, University of Tromsø, The Arctic University of Norway, Tromsø, Norway; ∗∗∗ Danish Cancer Society Research Center, Copenhagen, Denmark; ‡‡‡ Department of Public Health, Aarhus University, Aarhus, Denmark; §§§ Unit of Nutrition, Environment and Cancer, Cancer Epidemiology Research Programme, Catalan Institute of Oncology, L′Hospitallet de Llobregat, Barcelona, Spain; ‖‖‖ Facultat de Ciències de la Salut Blanquerna, Universitat Ramon Llull, Barcelona, Spain; ¶¶¶ Escuela Andaluza de Salud Pública, Instituto de Investigación Biosanitaria, Hospitales Universitarios de Granada/Universidad de Granada, Granada, Spain; ### Centro de Investigación Biomédica en Red de Epidemiología y Salud Pública, Madrid, Spain; ∗∗∗∗ Public Health Division of Gipuzkoa, Research Institute of BioDonostia, San Sebastian, Spain; ‡‡‡‡ Department of Epidemiology, Murcia Regional Health Council, Biomedical Research Institute of Murcia-Arrixaca, Murcia, Spain; §§§§ Navarra Public Health Institute, Pamplona, Spain; ‖‖‖‖ Navarra Institute for Health Research, Pamplona, Spain; ¶¶¶¶ Public Health Directorate, Asturias, Spain; #### University of Cambridge School of Clinical Medicine, Clinical Gerontology Unit, Addenbrooke’s Hospital, Cambridge, United Kingdom; ∗∗∗∗∗ Medical Research Council Epidemiology Unit, University of Cambridge School of Clinical Medicine, Cambridge, United Kingdom; ‡‡‡‡‡ Cancer Epidemiology Unit, Nuffield Department of Population Health, University of Oxford, Oxford, United Kingdom; §§§§§ Hellenic Health Foundation, Athens, Greece; ‖‖‖‖‖ World Health Organization Collaborating Center for Nutrition and Health, Unit of Nutritional Epidemiology and Nutrition in Public Health, Department of Hygiene, Epidemiology and Medical Statistics, School of Medicine, National and Kapodistrian University of Athens, Athens, Greece; ¶¶¶¶¶ Department of Clinical Sciences and Community Health, Università degli Studi di Milano, Milan, Italy; ##### Pulmonary Medicine Department, School of Medicine, National and Kapodistrian University of Athens, Attikon University Hospital, Haidari, Greece; ∗∗∗∗∗∗ Cancer Risk Factors and Life-Style Epidemiology Unit, Cancer Research and Prevention Institute, Cancer Research and Prevention Institute, Florence, Italy; ‡‡‡‡‡‡ Epidemiology and Prevention Unit, Fondazione Istituto di Ricovero e Cura a Carattere Scientifico Istituto Nazionale dei Tumori, Milan, Italy; §§§§§§ Cancer Registry and Histopathology Department, Civic-M.P. Arezzo Hospital, Azienda Sanitaria Provinciale Ragusa, Italy; ‖‖‖‖‖‖ Unit of Cancer Epidemiology, Department of Medical Sciences, University of Turin, Turin, Italy; ¶¶¶¶¶¶ Dipartimento di Medicina Clinica e Sperimentale, Federico II University, Naples, Italy; ###### Department for Determinants of Chronic Diseases, National Institute for Public Health and the Environment (Netherlands National Institute for Public Health and the Environment), Bilthoven, The Netherlands; ∗∗∗∗∗∗∗ Department of Gastroenterology and Hepatology, University Medical Centre, Utrecht, The Netherlands; §§§§§§§ Department of Epidemiology, Julius Center for Health Sciences and Primary Care, University Medical Centre, Utrecht, The Netherlands; ‡‡‡‡‡‡‡ Department of Social and Preventive Medicine, Faculty of Medicine, University of Malaya, Kuala Lumpur, Malaysia; ‖‖‖‖‖‖‖ Department of Medical Biosciences, Pathology, Umeå University, Umeå, Sweden; ¶¶¶¶¶¶¶ Department of Radiation Sciences, Oncology, Umeå University, Umeå, Sweden; ####### Division of Oncology and Pathology, Department of Clinical Sciences, Lund University, Lund, Sweden; ∗∗∗∗∗∗∗∗ Albert Einstein College of Medicine, Bronx, New York, New York

**Keywords:** Colorectal Cancer, Risk Factors, Anatomic Subsite, Heterogeneity, Proximal Colon, Distal Colon, Rectum, BMI, body mass index, CRC, colorectal cancer, EPIC, European Prospective Investigation into Cancer and Nutrition, HR, hazard ratio, MHT, menopausal hormone therapy, NSAID, nonsteroidal anti-inflammatory drug

## Abstract

**Background & Aims:**

Colorectal cancer located at different anatomical subsites may have distinct etiologies and risk factors. Previous studies that have examined this hypothesis have yielded inconsistent results, possibly because most studies have been of insufficient size to identify heterogeneous associations with precision.

**Methods:**

In the European Prospective Investigation into Cancer and Nutrition study, we used multivariable joint Cox proportional hazards models, which accounted for tumors at different anatomical sites (proximal colon, distal colon, and rectum) as competing risks, to examine the relationships between 14 established/suspected lifestyle, anthropometric, and reproductive/menstrual risk factors with colorectal cancer risk. Heterogeneity across sites was tested using Wald tests.

**Results:**

After a median of 14.9 years of follow-up of 521,330 men and women, 6291 colorectal cancer cases occurred. Physical activity was related inversely to proximal colon and distal colon cancer, but not to rectal cancer (*P* heterogeneity = .03). Height was associated positively with proximal and distal colon cancer only, but not rectal cancer (*P* heterogeneity = .0001). For men, but not women, heterogeneous relationships were observed for body mass index (*P* heterogeneity = .008) and waist circumference (*P* heterogeneity = .03), with weaker positive associations found for rectal cancer, compared with proximal and distal colon cancer. Current smoking was associated with a greater risk of rectal and proximal colon cancer, but not distal colon cancer (*P* heterogeneity = .05). No heterogeneity by anatomical site was found for alcohol consumption, diabetes, nonsteroidal anti-inflammatory drug use, and reproductive/menstrual factors.

**Conclusions:**

The relationships between physical activity, anthropometry, and smoking with colorectal cancer risk differed by subsite, supporting the hypothesis that tumors in different anatomical regions may have distinct etiologies.

**What You Need to Know**

**Background**

Previous research has indicated that colorectal tumors located at different anatomic sites have distinct clinical and molecular characteristics. It also has been hypothesized that colorectal cancer at different anatomic locations may have differential etiologies and risk factors. Previous epidemiologic studies may have been underpowered to detect heterogeneous relationships by anatomic site.

**Findings**

This was a large study that was performed to comprehensively investigate the relationships between colorectal cancer risk factors by anatomic site in both men and women, with more than 520,000 participants from 10 European countries included, and more than 6200 incident colorectal cancer cases. We found heterogeneous relationships across tumors located in the proximal colon, distal colon, and rectum for physical activity levels, anthropometric measurements, and smoking.

**Implications for patient care**

These results highlight the importance of separating the colorectum into distinct entities with separate etiologies. Variability in the carcinogenic processes at different sites of the large bowel may explain the complex risk factor–colorectal cancer relationships.

Colorectal cancer (CRC) is one of the most frequently occurring malignancies worldwide. In 2018, 1.8 million colorectal cancer diagnoses and 881,000 deaths are estimated to occur.^1^ Colorectal tumors at different anatomic sites have variable clinical characteristics.^2^ In the proximal colon, tumors typically present at a later stage with a poorer prognosis than those in the distal colon and rectum.^3^, ^4^ Women are more likely to develop cancers in the proximal colon, whereas in men cancers are more common in the distal colon region.^5^ In addition, with advancing age, a greater proportion of colorectal tumors are located in the proximal colon, with a reduced proportion of rectal tumors.^6^

Molecular heterogeneity also has been found for CRC tumors across anatomic sites. CpG island methylator phenotype–high, microsatellite instability–high, and *PIK3CA* and *BRAF* mutations are found most commonly in the proximal colon region, with a linear decrease in frequency across the distal colon and rectum regions.^7^ *KRAS* mutations have been found to be most common in the cecum region of the proximal colon, compared with other bowel regions.^7^ *TP53* mutations are more frequent in tumors in the distal colon and rectum, compared with the proximal colon.^8^, ^9^

CRC tumors at different anatomic locations also may have differential etiologies and risk factors.^6^, ^8^, ^10^, ^11^ Previous studies that have examined this hypothesis have yielded inconsistent results, possibly because most have been of insufficient size to identify heterogeneous associations with precision. We therefore performed a comprehensive investigation of how 14 established or suspected lifestyle, anthropometric, and reproductive and menstrual risk factors are associated with tumors located at the 3 main anatomic sites (proximal colon, distal colon, and rectum) in the European Prospective Investigation into Cancer and Nutrition (EPIC) cohort, with more than 520,000 participants. The large number of incident CRC cases (>6200) affords high statistical power to compare risk factor associations across tumor anatomic sites.

## Methods

### Study Population

EPIC is a multicenter prospective cohort of 521,448 participants, most were age 35 years and older, who were recruited between 1992 and 2000, predominantly from the general population of 10 European countries (Denmark, France, Germany, Greece, Italy, The Netherlands, Norway, Spain, Sweden, and the United Kingdom).^12^ Written informed consent was provided by all study participants, and ethical approval for EPIC was provided by the International Agency for Research on Cancer and local participating centers. Participants with cancer diagnoses before recruitment (n = 29,456); those in the highest and lowest 1% of the distribution for the ratio of energy intake to estimated energy requirement (n = 9573); and those with missing information on alcohol consumption and follow-up evaluation (n = 6259) were excluded from analyses. Additional exposure-specific exclusions were applied when there was missing information for the risk factor of interest.

### Exposures

The 14 CRC risk factors, all measured at recruitment, considered in the current analysis were as follows: alcohol consumption (per 15 g/d); ever nonsteroidal anti-inflammatory drug (NSAID) use (no, yes); physical activity index (inactive, moderately inactive, moderately active, active); prevalent diabetes (no, yes); smoking status (never, former, current); body mass index (BMI) (per 5 kg/m^2^); height (per 10 cm); waist circumference (per 5 cm); waist-to-hip ratio (per 0.05); and, in women only, age at menarche (<12, 12–13, 14–15, ≥15 y), age at menopause (≤50, 51–52, 53–54, ≥55 y); ever OC use (never, ever); ever menopausal hormone therapy (MHT) use (never, ever); and duration of MHT use (never users, <2, 2 to <5, 5 to <8, ≥8 y). In secondary analyses, we investigated the relationships by anatomic subsite for alcohol consumption from wine (per 15 g/d), beer (per 15 g/d), and spirits liquors (per 3 g/d). Full details of measurements are detailed in the Supplementary Methods section.

### Follow-Up Evaluation for Cancer Incidence and Vital Status

Cancer incidence was determined through record linkage with regional cancer registries or via a combination of methods, including the use of health insurance records, contacts with cancer and pathology registries, and active follow-up evaluation. CRC cases were defined using the 10th Revision of the International Classification of Diseases and the 2nd Revision of the International Classification of Diseases for Oncology. Proximal colon cancer included those tumors within the cecum, appendix, ascending colon, hepatic flexure, transverse colon, and splenic flexure (C18.0–18.5). Distal colon cancer included those within the descending (C18.6) and sigmoid (C18.7) colon. Cancer of the rectum included cancer occurring at the rectosigmoid junction (C19) and rectum (C20).

### Statistical Analysis

Hazard ratios (HRs) and the corresponding 95% CIs for the 14 risk factors and CRC were estimated using Cox proportional hazards models. Age was used as the time-scale in all models. Time at entry was age at recruitment. Exit time was age at whichever of the following came first: CRC diagnosis, death, or the last date at which follow-up evaluation was considered complete in each center. For the analyses by anatomic site, HRs and 95% CIs were estimated using a multivariable joint Cox proportional hazards model, which accounted for tumors located at different anatomic sites as competing risks.^13^ Heterogeneity across sites was tested using Wald tests. Full details on the statistical methods are shown in the Supplementary Methods section and are detailed by Xue et al.^13^ Separate models were run for body size measurements and CRC for men and women because of a priori knowledge that the relationship differs by sex.^14^ To determine whether the lifestyle risk factors and CRC relationships differed by sex, we included an interaction term for sex (multiplicative scale) in the model. The statistical significance of the cross-product term was evaluated using the likelihood ratio test. Because no heterogeneity was found by sex for smoking status (*P* interaction = .36), physical activity (*P* interaction = .71), alcohol consumption (*P* interaction = .45), diabetes (*P* interaction = .83), or NSAID use (*P* interaction = .34), men and women were analyzed together. Multivariable models were, where appropriate, mutually adjusted. We also conducted sensitivity analyses separating tumors located in the cecum (C18) into an additional anatomic site and examining heterogeneity in the relationships to each risk factor across 4 anatomic sites (cecum colon vs proximal colon vs distal colon vs rectum). Statistical tests used in the analysis all were 2-sided and a *P* value less than .05 was considered statistically significant.

## Results

During a median follow-up period of 14.9 years, 6291 CRC cases occurred (2718 in men and 3573 in women). Of these, 1877 were located in the proximal colon, 1743 in the distal colon, and 2094 in the rectum. Table 1 shows the characteristics of participants included in the analysis.

**Table 1:** Characteristics of Participants at Recruitment

	Both sexes				
	Non-cases	Colorectal cancer cases	Colon proximal cancer cases	Colon distal cancer cases	Rectal cancer cases
*N*	469,869	6291	1877	1743	2094
Women, *%*	70.3	56.8	64.4	56.0	50.7
Age at recruitment, *y*	51.2 (9.9)	57.3 (7.9)	58.2 (7.9)	56.9 (7.5)	56.6 (7.7)
Alcohol consumption, *g/d*	11.6 (16.8)	15.0 (20.2)	12.6 (18.4)	15.4 (20.5)	16.5 (21.4)
Smoking status					
Never, *%*	49.1	40.7	43.6	40.4	38.4
Current, *%*	22.4	24.1	22.8	22.3	26.0
Ever NSAID use					
Yes, *%*	8.2	8.5	8.2	9.4	8.3
Physical activity					
Inactive, *%*	20.9	24.9	27.9	25.0	21.8
Active, *%*	17.9	18.4	15.6	18.7	21.4
Prevalent diabetes					
Yes, *%*	2.8	4.4	4.5	4.6	3.8
Body mass index, *kg/m*^*2*^					
Men	26.5 (3.6)	27.2 (3.8)	27.3 (4.0)	27.5 (3.8)	26.9 (3.6)
Women	25.4 (4.6)	26.1 (4.6)	25.9 (4.5)	26.3 (4.7)	26.0 (4.5)
Height, *cm*					
Men	174.7 (7.4)	174.4 (7.1)	175.2 (7.1)	174.5 (7.3)	174.2 (7.0)
Women	161.8 (6.8)	161.8 (6.6)	162.3 (6.2)	161.7 (6.6)	161.5 (6.4)
Waist circumference, *cm*					
Men	94.6 (10.2)	97.4 (10.2)	97.6 (10.4)	98.2 (10.5)	96.8 (9.9)
Women	80.2 (11.5)	82.6 (11.7)	82.6 (11.5)	83.1 (12.1)	82.0 (11.7)
Waist-to-hip ratio					
Men	0.94 (0.1)	0.96 (0.1)	0.95 (0.1)	0.96 (0.1)	0.96 (0.1)
Women	0.79 (0.1)	0.81 (0.1)	0.81 (0.1)	0.81 (0.1)	0.80 (0.1)
Age at menarche, *y*	13.1 (1.5)	13.2 (1.6)	13.2 (1.6)	13.2 (1.6)	13.2 (1.5)
Age at menopause, *y*	48.6 (5.0)	49.0 (5.0)	49.0 (5.0)	49.0 (4.8)	49.2 (5.1)
Ever oral contraceptive use					
Yes, *%*	58.8	47.5	45.3	48.2	51.9
Ever MHT use					
Yes, *%*	25.9	31.1	32.8	29.5	30.9
Education					
Longer education (including university)	24.2	19.0	19.1	18.4	18.8
Red and processed meat intake, *g/d*	74.7 (51.0)	83.0 (52.7)	78.8 (51.3)	82.7 (52.3)	87.2 (53.5)
Calcium intake, *mg/d*	994.8 (409.4)	985.0 (398.5)	994.1 (392.6)	970.4 (393.6)	984.2 (401.3)
Fiber intake, *g/d*	22.8 (7.7)	22.6 (7.7)	22.5 (7.6)	22.5 (7.9)	22.8 (7.5)

NOTE. Based on participant numbers in the alcohol consumption models. Means and SD are shown unless stated otherwise.

MHT, menopausal hormone therapy; NSAID, nonsteroidal anti-inflammatory drug.

**Figure 1:**
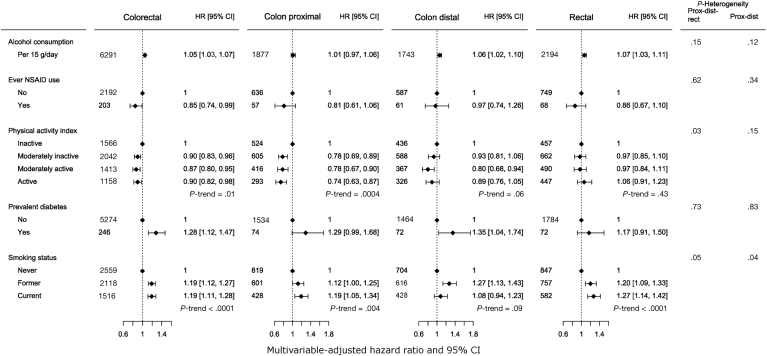
Multivariable-adjusted HRs and 95% CIs for colorectal cancer incidence for both sexes combined in relation to lifestyle factors, by anatomic site. For alcohol consumption, physical activity, and smoking status: multivariable models–Cox regression using age as the underlying time variable and stratified by sex, center, and age at recruitment. Models mutually adjusted, and additionally adjusted for body mass index, height, education level, ever use of menopausal hormone therapy, and intakes of alcohol, red and processed meats, calcium, and fiber. For ever NSAID use and prevalent diabetes: multivariable models–Cox regression using age as the underlying time variable and stratified by sex, center, and age at recruitment adjusted for body mass index, height, physical activity; smoking status and intensity; education level; ever use of menopausal hormone therapy; and intakes of alcohol, red and processed meats, calcium, and fiber. Information on NSAID use was available from only 6 centers: Cambridge, Utrecht, Heidelberg, Potsdam, Aarhus, and Copenhagen. Prox-dist-rect, proximal, distal, rectal.

Alcohol consumption, prevalent diabetes, and smoking were associated with a greater risk of CRC, and ever NSAID use and physical activity were associated with a lower risk (Figure 1). For physical activity, compared with being inactive, the physically active group had a lower risk of developing CRC (HR, 0.90; 95% CI, 0.82–0.98; *P* trend = .01). This inverse association was most evident for proximal colon cancers (HR, 0.74; 95% CI, 0.63–0.87; *P* trend = .0004), although the estimates were not statistically significant for distal colon or rectal cancers (*P* heterogeneity for proximal-distal-rectal = .03). Smoking was associated with the development of CRC (current smokers vs never smokers: HR, 1.19; 95% CI, 1.11–1.28; *P* trend < .0001). By anatomic site, heterogeneity was observed, with current smoking (vs never smokers) associated with increased risks of proximal colon cancer (HR, 1.19; 95% CI, 1.05–1.34) and rectal cancer (HR, 1.27; 95% CI, 1.14–1.42), but not distal colon cancer (HR, 1.08; 95% CI, 0.94–1.23) (*P* heterogeneity across 3 sites = .05; *P* heterogeneity for proximal and distal colon = .04). Former smoking was associated with a greater risk of developing distal colon cancer (vs never smokers: HR, 1.27; 95% CI, 1.13–1.43). Greater alcohol consumption was associated with an increased risk of CRC (per 15-g/d increment: HR, 1.05; 95% CI, 1.03–1.07). Although the test for heterogeneity was not statistically significant (*P* heterogeneity = .15 for proximal-distal-rectal), positive associations were found for distal colon and rectal cancers, but not for proximal colon cancer. No heterogeneity was observed for tumors located at different anatomic subsites for alcohol from wine, beer, and spirits/liquors when analyzed separately (all *P* heterogeneity > .05) (Supplementary Table 1). Prevalent diabetes at baseline (yes vs no) was associated with a higher CRC risk (HR, 1.28; 95% CI, 1.12–1.47), with similar positive relationships found across anatomic sites (*P* heterogeneity > .70), although the association for rectal cancer was not statistically significant. Ever use of NSAIDs was associated with a lower CRC risk (vs never use: HR, 0.85; 95% CI, 0.74–0.99), with no heterogeneity observed for tumors located at different anatomic sites (all *P* heterogeneity > .30).

**Figure 2:**
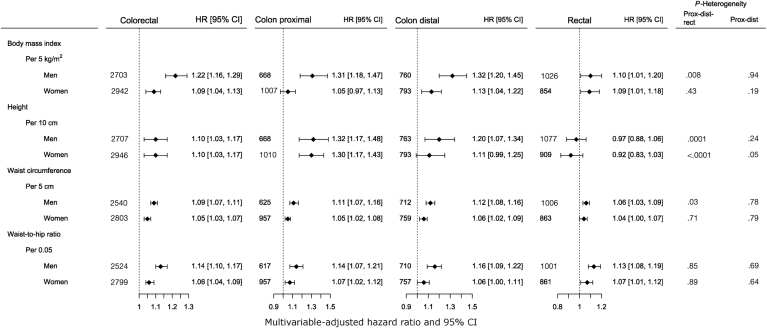
Multivariable-adjusted HRs and 95% CIs for colorectal cancer incidence for both sexes combined in relation to anthropometric measures, by anatomic site. Multivariable models only–Cox regression using age as the underlying time variable and stratified by center and age at recruitment, and adjusted for physical activity, smoking status and intensity, education level, ever use of menopausal hormone therapy, and intakes of alcohol, red and processed meats, calcium, and fiber. Multivariable model for height was adjusted further for body mass index. Multivariable models for body mass index, waist circumference, and waist-to-hip ratio were adjusted further for height. Prox-dist-rect, proximal, distal, rectal.

For men and women, higher BMI, height, waist circumference, and waist-to-hip ratio all were associated with a greater risk of CRC (Figure 2). For men, the positive relationship for BMI was weaker for rectal cancer (per 5 kg/m^2^: HR, 1.10; 95% CI, 1.01–1.20), compared with proximal colon cancer (per 5 kg/m^2^: HR, 1.31; 95% CI, 1.18–1.47) and distal colon cancer (per 5 kg/m^2^: HR, 1.32; 95% CI, 1.20–1.45) (*P* heterogeneity = .008), but no heterogeneity was found between tumors in the proximal and distal colon (*P* heterogeneity = .94). In addition, in men, the positive waist circumference association was weaker for tumors located in the rectum (per 5 cm: HR, 1.06; 95% CI, 1.03–1.09), than for tumors in the proximal colon (per 5 cm: HR, 1.11; 95% CI, 1.07–1.16) and distal colon (per 5 cm: HR, 1.12; 95% CI, 1.08–1.16) (*P* heterogeneity = .03), but no heterogeneity was found across the colon (proximal vs distal *P* heterogeneity = .78). The positive association between the waist-to-hip ratio and CRC for men and women was consistent across all anatomic sites (all *P* heterogeneity > .60). For men and women, height was not associated with rectal cancer (per 10 cm in men: HR, 0.97; 95% CI, 0.88–1.06; per 10 cm in women: HR, 0.92; 95% CI, 0.83–1.03), but was related positively to both proximal colon and distal colon cancers (*P* heterogeneity = .0001 for men and *P* heterogeneity < .0001 for women). The association of height with colon cancer did not differ between the proximal and distal colon in men (*P* heterogeneity = .24), but there was some suggestion of heterogeneity for women (*P* heterogeneity = .05), with a stronger positive association observed for proximal colon cancer (per 10 cm: HR, 1.30; 95% CI, 1.17–1.43) than for distal colon cancer (per 10 cm: HR, 1.11; 95% CI, 0.99–1.25). For women, no heterogeneity by subsite was observed for the other anthropometric measurements, with similar strength associations found for BMI, waist circumference, and waist-to-hip ratio across tumors at the 3 anatomic sites (all *P* heterogeneities > .05).

**Figure 3:**
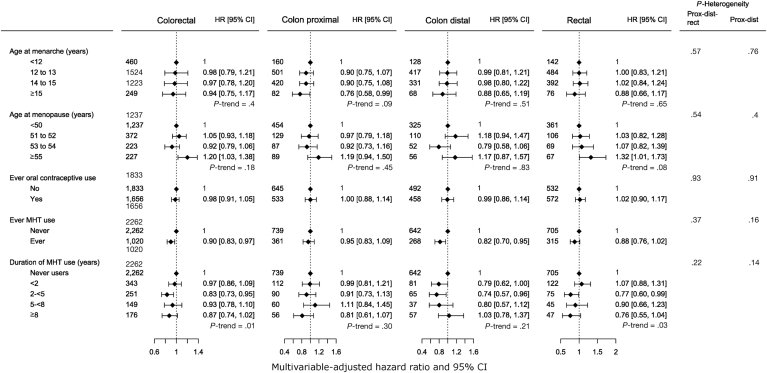
Multivariable-adjusted HRs and 95% CIs for colorectal cancer incidence in relation to reproductive and menstrual factors among women, by anatomic site. Multivariable models only–Cox regression using age as the underlying time variable and stratified by center and age at recruitment, and adjusted for body mass index, height, physical activity, smoking status and intensity, education level, ever use of menopausal hormone therapy, and intakes of alcohol, red and processed meats, calcium, and fiber. Prox-dist-rect, proximal, distal, rectal.

Ever MHT use vs never use was associated with a lower risk of CRC (HR, 0.90; 95% CI, 0.83–0.97), with no evidence of heterogeneity across subsites (*P* heterogeneity > .16) (Figure 3). The duration of MHT use was associated inversely with CRC risk (*P* trend = .01), with no heterogeneity found by anatomic site (*P* heterogeneity > .05). Age at menarche and ever OC use was not associated with CRC and no heterogeneity was observed across anatomic sites (*P* heterogeneity > .05). Older age (≥55 y) vs younger age at menopause (≤50 y) was associated with increased CRC risk (HR, 1.20; 95% CI, 1.03–1.38), with similar relationships observed by anatomic site (*P* heterogeneity > .40).

When tumors located in the cecum were considered as an additional subsite end point, a similar pattern of heterogeneous relationships was considered across the 4 subsites (cecum colon, proximal colon, distal colon, and rectum) (Supplementary Table 2, Supplementary Table 3, Supplementary Table 4).

## Discussion

In this multicountry prospective study, we found heterogeneous relationships by tumor site for physical activity, smoking, and anthropometric measurements. Low levels of physical activity and greater height and BMI were associated primarily with an increased risk of distal or proximal colon cancer, with weaker or null relationships found for rectal cancer. Current smoking was associated with an increased risk of proximal colon and rectal cancer, whereas no heterogeneity by anatomic site was found for alcohol consumption, prevalent diabetes, NSAID use, and, in women, reproductive and menstrual factors.

For overall CRC, we observed the expected pattern of risk factor associations. Greater adiposity and height were associated with increased CRC risk, as were higher alcohol consumption, smoking, prevalent diabetes, and later age at menopause. Conversely, being physically active and use of NSAIDs and MHT were associated with a lower risk of developing CRC. Our analysis benefited from the large number of incident CRC cases that accrued during the longer follow-up period, which allowed well-powered analyses for the 14 risk factors by tumor anatomic site. Recently, a similar analysis of CRC risk factors by anatomic site was performed in a large UK cohort, with no heterogeneity found for the considered risk factors by tumor anatomic site^15^; however, that study included only women, so it is uncertain whether the findings are generalizable to men.^15^ Previous studies that have investigated heterogeneity in the association between major risk factors and colorectal anatomic subsites in men and women had smaller numbers of cases compared with our analysis, and may have been constrained by insufficient statistical power to identify weak-to-moderate strength heterogeneous associations.^16^, ^17^ In the current study, which included men and women, we observed heterogeneous relationships between several risk factors and tumors across different anatomic sites.

We found that greater physical activity was related similarly to lower risks of developing tumors in the proximal and distal colon regions, findings consistent with other large prospective studies,^15^, ^17^ and a meta-analysis of 21 studies.^18^ Physical activity, however, was not related to rectal cancer risk, a result inconsistent with a recent participant-level pooled analysis that reported an inverse relationship between physical activity and rectal cancer incidence,^19^ but in accordance with a joint Nurses’ Health Study and Health Professionals Follow-up Study analysis.^10^ The biological mechanisms through which physical activity potentially decreases colon cancer risk, but not rectal cancer risk, are uncertain. Being physically active is associated with less weight gain and body fatness,^20^ and therefore has a beneficial effect on CRC risk.^21^ However, in our study, we found that greater BMI and waist circumference were risk factors for colon and, albeit more weakly, for rectal cancer. Greater physical activity also has been associated with lower insulin levels and beneficial effects on inflammatory pathways and dyslipidemia, including decreasing levels of circulating triglycerides.^22^, ^23^, ^24^ Previous meta-analyses have suggested that C-peptide (a marker of insulin secretion), C-reactive protein (a nonspecific marker of systemic inflammation), and triglycerides are associated positively with colon, but not rectal, cancer.^25^, ^26^, ^27^, ^28^ This suggests that any beneficial effects of physical exercise on insulin (or correlated metabolic markers), inflammatory, and lipid pathways would be more likely to influence tumors in the colon, and not in the rectum, potentially explaining the null result we observed for physical activity with rectal cancer.

Our finding that higher BMI was related more strongly to greater CRC risk among men than among women is in accordance with a large body of epidemiologic evidence.^21^, ^29^, ^30^ We observed heterogeneous relationships for anthropometric measurements by anatomic site, particularly for men. For BMI, the positive relationship found among men was weaker for rectal cancer compared with tumors in the colon. A meta-analysis of prospective studies also observed that, for men, a greater BMI was associated more weakly with rectal cancer (relative risk per 5-kg/m^2^ unit increase in BMI, 1.12; 95% CI, 1.09–1.16) than with colon cancer (relative risk per 5-kg/m^2^ unit increase in BMI, 1.30; 95% CI, 1.25–1.35).^21^ A moderately weaker positive relationship was found for waist circumference and rectal cancer in men compared with colonic subsites, however, for waist-to-hip ratio no heterogeneity by anatomic site was observed. For men and women, height was associated with colon cancer, but not with rectal cancer. This null result for rectal cancer is inconsistent with other large prospective cohort studies and a meta-analysis that found a positive association for height and rectal cancer.^31^, ^32^ In addition, positive relationships of similar magnitude were found for both colon and rectal cancer in a Mendelian randomization analysis.^33^

Current smoking was related to an increased risk of proximal colon and rectal cancers, but not distal colon cancer. A similar pattern of results for smoking history was found in the Nurses’ Health Study, with 40 pack-years of smoking (vs none) being associated positively only with proximal colon (HR, 1.31; 95% CI, 1.16–1.48) and rectal cancer (HR, 1.27; 95% CI, 1.05–1.53), but not distal colon cancer (HR, 1.04; 95% CI, 0.88–1.23).^17^ Microsatellite instability–high, *BRAF* mutation–positive, and CpG island methylator phenotype–positive tumors, are more common in the proximal colon region compared with the distal colon,^7^ and have been associated positively with cigarette smoking.^11^ However, these molecular characteristics are even less common for malignant tumors in the rectum, the subsite for which we observed the strongest positive relationship with smoking. In addition, a positive relationship was observed for former smokers and distal colon cancer, which is inconsistent with these molecular characteristics explaining these findings.

The current investigation was a large study that comprehensively investigated the relationships between CRC risk factor by anatomic site in both men and women. Limitations of our analysis were that all of the considered risk factors were measured once at baseline, and because of multiple known or suspected CRC risk factors being investigated simultaneously, some of our results could have been chance findings. Finally, our study would have been enhanced with information on tumor molecular features.

In conclusion, heterogeneous relationships across tumors located in the proximal colon, distal colon, and rectum were observed for physical activity, anthropometric measurements, and smoking. These results, taken together with the varying biological and molecular features of tumors located across the colorectum, indicate that tumors in different anatomic regions may have distinct etiologies.

## Figures and Tables

**Supplementary Table 1:** Multivariable-Adjusted HRs and 95% CIs for Colorectal Cancer Incidence for Both Sexes Combined in Relation to Alcohol Intake (Overall and by Source), by Anatomic Site

	Colorectal cancer		Colon proximal		Colon distal		Rectal	
	N cases	Multivariable	N cases	Multivariable	N cases	Multivariable	N cases	Multivariable
Alcohol								
Per 15 g/d	6291	1.05 (1.03–1.07)	1877	1.01 (0.97–1.06)	1743	1.06 (1.02–1.10)	2094	1.07 (1.03–1.11)
* P* heterogeneity proximal-distal-rectal	.15							
* P* heterogeneity proximal-distal	.12							
Alcohol from wine								
Per 15 g/d	6291	1.03 (0.99–1.06)	1877	1.00 (0.93–1.07)	1743	1.05 (1.00–1.11)	2094	1.04 (0.99–1.09)
* P* heterogeneity proximal-distal-rectal	.46							
* P* heterogeneity proximal-distal	.22							
Alcohol from beer								
Per 15 g/d	6291	1.09 (1.05–1.13)	1877	1.03 (0.94–1.12)	1743	1.10 (1.03–1.17)	2094	1.11 (1.06–1.16)
* P* heterogeneity proximal-distal-rectal	.29							
* P* heterogeneity proximal-distal	.21							
Alcohol from spirits/liquors								
Per 3 g/d	6291	1.01 (1.00–1.03)	1877	1.00 (0.97–1.04)	1743	1.00 (0.96–1.03)	2094	1.02 (1.00–1.05)
* P* heterogeneity proximal-distal-rectal	.27							
* P* heterogeneity proximal-distal	.80							

NOTE. Multivariable models only: Cox regression used age as the underlying time variable and was stratified by sex, center, and age at recruitment. Models were adjusted for body mass index, height, physical activity index, smoking status and intensity, education level attained, ever use of menopausal hormone therapy, and intakes of red and processed meats, dietary calcium, and fiber.

HRs, hazard ratios.

**Supplementary Table 2:** Multivariable-Adjusted HRs and 95% CIs for Colorectal Cancer Incidence for Both Sexes Combined in Relation to Lifestyle Factors, by Tumors in the Colon Cecum, Colon Proximal, Colon Distal, and Rectum

	Colon cecum		Colon proximal		Colon distal		Rectal	
	N cases	Multivariable	N cases	Multivariable	N cases	Multivariable	N cases	Multivariable
Alcohol consumption								
Per 15 g/d	720	1.00 (0.92–1.09)	1198	1.03 (0.97–1.08)	1743	1.06 (1.02–1.10)	2211	1.07 (1.04–1.11)
* P* heterogeneity cecum-proximal-distal-rectal	.33							
Ever NSAID use^a^								
No	257	1	587	1	587	1	802	1
Yes	28	0.91 (0.61–1.35)	61	0.73 (0.50–1.05)	61	0.97 (0.74–1.26)	73	0.86 (0.67–1.09)
* P* heterogeneity cecum-proximal-distal-rectal	.67							
Physical activity index								
Inactive	196	1	344	1	436	1	457	1
Moderately inactive	231	0.88 (0.72–1.09)	383	0.72 (0.62–0.84)	588	0.93 (0.81–1.06)	662	0.97 (0.86–1.10)
Moderately active	156	0.88 (0.69–1.13)	270	0.73 (0.60–0.87)	367	0.80 (0.69–0.94)	490	0.99 (0.87–1.15)
Active	113	0.83 (0.64–1.08)	186	0.68 (0.56–0.83)	326	0.90 (0.76–1.05)	447	1.07 (0.93–1.24)
* P* trend		.18		.0003		.06		.29
* P* heterogeneity cecum-proximal-distal-rectal	.02							
Prevalent diabetes								
No	559	1	1012	1	1464	1	1784	1
Yes	23	1.29 (0.84–2.00)	54	1.33 (0.97–1.82)	72	1.34 (1.04–1.74)	72	1.21 (0.95–1.54)
* P* heterogeneity cecum-proximal-distal-rectal	.94							
Smoking status								
Never	320	1	509	1	704	1	847	1
Former	233	1.07 (0.89–1.27)	385	1.18 (1.03–1.36)	616	1.27 (1.13–1.43)	757	1.20 (1.09–1.33)
Current	151	1.12 (0.91–1.38)	289	1.25 (1.08–1.46)	388	1.08 (0.94–1.23)	582	1.27 (1.14–1.42)
* P* trend		.27		.0017		.09		<.0001
* P* heterogeneity cecum-proximal-distal-rectal	.13							

NOTE. For alcohol consumption, physical activity index, and smoking status: multivariable models only, Cox regression used age as the underlying time variable and was stratified by sex, center, and age at recruitment. Models were mutually adjusted, and additionally adjusted for body mass index, height, education level attained, ever use of menopausal hormone therapy, and intakes of alcohol, red and processed meats, dietary calcium, and fiber. For ever NSAID use and prevalent diabetes: multivariable models only, Cox regression used age as the underlying time variable and was stratified by sex, center, and age at recruitment adjusted for body mass index, height, physical activity index; smoking status and intensity; education level attained; ever use of menopausal hormone therapy; and intakes of alcohol, red and processed meats, dietary calcium, and fiber.

HR, hazard ratio, NSAID, nonsteroidal anti-inflammatory drug.

a Information on NSAID use was available from only 6 centers (Cambridge, UK; Utrecht, The Netherlands; Heidelberg and Potsdam, Germany; Aarhus and Copenhagen, Denmark).

**Supplementary Table 3:** Multivariable-Adjusted HRs and 95% CIs for Colorectal Cancer Incidence for Both Sexes Combined in Relation to Anthropometric Measures, by Tumors in the Colon Cecum, Colon Proximal, Colon Distal, and Rectum

	Colon cecum		Colon proximal		Colon distal		Rectal	
	N cases	Multivariable	N cases	Multivariable	N cases	Multivariable	N cases	Multivariable
BMI								
Men								
Per 5 kg/m^2^	250	1.41 (1.19–1.68)	437	1.26 (1.09–1.45)	760	1.32 (1.20–1.45)	1076	1.11 (1.02–1.03)
* P* heterogeneity cecum-proximal-distal-rectal	.01							
Women								
Per 5 kg/m^2^	405	1.06 (0.94–1.19)	624	1.06 (0.97–1.16)	793	1.13 (1.04–1.22)	854	1.08 (1.01–1.16)
* P* heterogeneity cecum-proximal-distal-rectal	.72							
Height								
Men								
10 cm	250	1.43 (1.18–1.75)	437	1.22 (1.06–1.42)	763	1.20 (1.07–1.34)	1077	0.95 (0.86–1.04)
* P* heterogeneity cecum-proximal-distal-rectal	<.0001							
Women								
Per 10 cm	407	1.30 (1.11–1.52)	625	1.26 (1.11–1.45)	793	1.10 (0.99–1.25)	909	0.92 (0.83–1.03)
* P* heterogeneity cecum-proximal-distal-rectal	.0003							
Waist circumference								
Men								
Per 5 cm	236	1.13 (1.06–1.20)	409	1.10 (1.05–1.16)	712	1.12 (1.08–1.17)	1006	1.06 (1.03–1.09)
* P* heterogeneity cecum-proximal-distal-rectal	.05							
Women								
Per 5 cm	389	1.04 (0.99–1.09)	591	1.06 (1.02–1.10)	759	1.06 (1.02–1.09)	863	1.04 (1.00–1.07)
* P* heterogeneity cecum-proximal-distal-rectal	.78							
Waist-to-hip ratio								
Men								
Per 0.05	233	1.14 (1.04–1.25)	404	1.12 (1.04–1.21)	710	1.16 (1.09–1.22)	1001	1.13 (1.08–1.19)
* P* heterogeneity cecum-proximal-distal-rectal	.93							
Women								
Per 0.05	389	1.04 (0.96–1.13)	591	1.10 (1.04–1.16)	757	1.06 (1.00–1.11)	861	1.07 (1.01–1.12)
* P* heterogeneity cecum-proximal-distal-rectal	.62							

NOTE. Multivariable models only: Cox regression used age as the underlying time variable and was stratified by center and age at recruitment, and adjusted for physical activity index, smoking status and intensity, education level attained, ever use of menopausal hormone therapy, and intakes of alcohol, red and processed meats, dietary calcium, and fiber. Multivariable model for height was adjusted further for body mass index. Multivariable models for body mass index, waist circumference, and waist-to-hip ratio were adjusted further for height.

BMI, body mass index; HRs, hazard ratios.

**Supplementary Table 4:** Multivariable-Adjusted HRs and 95% CIs for Colorectal Cancer Incidence Among Women in Relation to Reproductive and Menstrual Characteristics, by Tumors in the Colon Cecum, Colon Proximal, Colon Distal, and Rectum

	Colon cecum		Colon proximal		Colon distal		Rectal	
	N cases	Multivariable	N cases	Multivariable	N cases	Multivariable	N cases	Multivariable
Age at menarche, *y*								
<12	13	1	23	1	28	1	22	1
12–13	144	0.92 (0.52–1.63)	205	0.75 (0.48–1.15)	276	0.89 (0.61–1.32)	325	1.30 (0.84–2.00)
14–15	203	0.77 (0.44–1.36)	348	0.75 (0.49–1.15)	460	0.92 (0.63–1.36)	545	1.34 (0.87–2.06)
≥15	92	0.78 (0.43–1.41)	157	0.79 (0.51–1.23)	180	0.88 (0.59–1.32)	202	1.21 (0.78–1.89)
*P* trend		.1372		.9997		.7919		.9427
*P* heterogeneity cecum-proximal-distal-rectal	.54							
Age at menopause, *y*								
≤50	172	1	287	1	325	1	361	1
51–52	53	1.06 (0.77–1.44)	78	0.93 (0.72–1.20)	110	1.18 (0.94–1.47)	106	1.03 (0.82–1.28)
53–54	31	0.84 (0.57–1.23)	59	1.02 (0.77–1.35)	52	0.79 (0.58–1.06)	69	1.07 (0.82–1.39)
≥55	46	1.52 (1.10–2.12)	48	1.05 (0.76–1.43)	56	1.17 (0.87–1.57)	67	1.32 (1.01–1.73)
*P* trend		.1281		.8442		.8376		.0794
*P* heterogeneity cecum-proximal-distal-rectal	.44							
Ever oral contraceptive use								
No	276	1	380	1	492	1	532	1
Yes	179	0.82 (0.66–1.01)	365	1.14 (0.97–1.34)	458	0.99 (0.86–1.14)	572	1.02 (0.90–1.17)
*P* heterogeneity cecum-proximal-distal-rectal	.11							
Ever menopausal hormone therapy use								
Never		1		1		1		1
Ever		0.90 (0.83–0.97)		0.95 (0.83–1.09)		0.82 (0.70–0.95)		0.88 (0.76–1.02)
*P* heterogeneity cecum-proximal-distal-rectal	.37							
Duration of menopausal hormone therapy use, *y*								
Never users	288	1	467	1	642	1	705	1
<2	46	1.08 (0.78–1.50)	68	0.93 (0.72–1.21)	81	0.79 (0.62–1.00)	122	1.07 (0.88–1.31)
2 to <5	34	0.94 (0.65–1.35)	60	0.92 (0.70–1.22)	65	0.74 (0.57–0.96)	75	0.77 (0.60–0.99)
5 to <8	21	1.00 (0.63–1.60)	39	1.12 (0.80–1.57)	37	0.79 (0.56–1.12)	45	0.90 (0.66–1.23)
≥8	23	0.76 (0.49–1.19)	34	0.82 (0.57–1.18)	57	1.03 (0.78–1.37)	47	0.76 (0.55–1.04)
*P* trend		.34		.46		.21		.03
*P* heterogeneity cecum-proximal-distal-rectal	.46							

NOTE. Multivariable models only: Cox regression used age as the underlying time variable and was stratified by center and age at recruitment, and adjusted for body mass index, height, physical activity index, smoking status and intensity, education level attained, ever use of menopausal hormone therapy, and intakes of alcohol, red and processed meats, dietary calcium, and fiber.

CI, confidence interval, HRs, hazard ratios.

## Supplementary Methods

### Exposures

The 14 colorectal cancer risk factors, all measured at recruitment, considered in the current analysis were as follows: alcohol consumption (per 15-g/d increment); ever NSAID use (no, yes); physical activity index (inactive, moderately inactive, moderately active, active); prevalent diabetes (no, yes); smoking status (never, former, current); BMI (per 5-kg/m^2^ increment); height (per 10-cm increment); waist circumference (per 5-cm increment); waist-to-hip-ratio (per 0.05 increment); and, in women only, age at menarche (<12, 12–13, 14–15, ≥15 y); age at menopause (≤50, 51–52, 53–54, ≥55 y); ever oral contraceptive use (never, ever); ever MHT use (never, ever); and duration of MHT use (never users, <2, 2 to <5, 5 to <8, ≥8 y). In secondary analyses, we investigated the relationships by anatomic subsite for alcohol consumption from wine (per 15-g/d increment), beer (per 15-g/d increment), and spirits liquors (per 3-g/d increment).

With participants not wearing shoes, weight was measured to the nearest 0.1 kg and height was measured—dependent on the study center—to the nearest 0.1, 0.5, or 1.0 cm. BMI was calculated as weight in kilograms divided by height in meters squared. Waist circumference was measured either at the narrowest torso circumference or at the midpoint between the lower ribs and iliac crest. Hip circumference was measured at the widest circumference (France; Italy; Spain; Bilthoven, The Netherlands; Greece; Malmö, Sweden) or over the buttocks (the United Kingdom; Utrecht, The Netherlands; Germany; Denmark). The waist-to-hip ratio was calculated by dividing the waist circumference by the hip circumference. Standardized lifestyle and personal history questionnaires were collected at recruitment,^1^, ^2^ before disease onset or diagnosis. Information on cigarette smoking habits included baseline smoking status (never, former, or current smoker). Overall physical activity (the sum/total of occupational physical activity and leisure time physical activity) was assessed from 3 questions referring to the past year and an index was derived by allocating individuals to 4 categories of overall activity (inactive, moderately inactive, moderately active, and active).^3^ Information was collected on education, diabetes prevalence, oral contraceptive use, MHT use, age at menarche, age at menopause, and, in 6 centers (Cambridge, UK; Utrecht, The Netherlands; Heidelberg and Potsdam, Germany; Aarhus and Copenhagen, Denmark), NSAID use (including aspirin). Diet over the previous 12 months was assessed at recruitment using validated country-/center-specific dietary questionnaires.^1^, ^2^ Alcohol consumption at recruitment was calculated from the number of standard glasses of beer, wine, cider, sweet liquor, distilled spirits, or fortified wines consumed per day/week reported during the 12 months before recruitment.

### Follow-Up Evaluation for Cancer Incidence and Vital Status

Cancer incidence was determined through record linkage with regional cancer registries (Denmark, Italy, The Netherlands, Norway, Spain, Sweden, and the United Kingdom) or via a combination of methods, including the use of health insurance records, contacts with cancer and pathology registries, and active follow-up evaluation through participants and their next of kin (France, Germany, and Greece). Colorectal cancer cases were defined using the 10th Revision of the International Classification of Diseases and the 2nd Revision of the International Classification of Diseases for Oncology. Proximal colon cancer included those within the cecum, appendix, ascending colon, hepatic flexure, transverse colon, and splenic flexure (C18.0–18.5). Distal colon cancer included those within the descending (C18.6) and sigmoid (C18.7) colon. Cancer of the rectum included cancer occurring at the rectosigmoid junction (C19) and rectum (C20).

### Statistical Analysis

HRs and the corresponding 95% CIs for the 14 risk factors and CRC were estimated using Cox proportional hazards models. Age was used as the time-scale in all models. Time at entry was age at recruitment. Exit time was age at whichever of the following came first: colorectal cancer diagnosis, death, or the last date at which follow-up evaluation was considered complete in each center. Possible nonproportionality was assessed using an analysis of Schoenfeld^4^ residuals, with no evidence of nonproportionality being detected. For the analyses by anatomic site, HRs and 95% CIs were estimated using multivariable joint Cox proportional hazards model, which accounted for tumors located at different anatomic sites as competing risks.^5^ The heterogeneity in baseline risk of colorectal cancer subsites was addressed by stratified Cox models, in which each subsite was allowed to have its own baseline hazard function; the heterogeneity in association with risk factors across subsites was assessed by including an interaction term between each risk factor and the indicators of colorectal cancer subsites and testing the statistical significance of the interaction terms. Because a robust variance was used to address the competing risk between colorectal cancer subsites, a log-likelihood ratio test was no longer valid. We therefore used a global Wald test based on the robust variance estimates obtained from a sandwich type of estimator.
